# A Novel Human Polycomb Binding Site Acts As a Functional Polycomb Response Element in *Drosophila*

**DOI:** 10.1371/journal.pone.0036365

**Published:** 2012-05-03

**Authors:** Suresh Cuddapah, Tae-Young Roh, Kairong Cui, Cynthia C. Jose, Margaret T. Fuller, Keji Zhao, Xin Chen

**Affiliations:** 1 Department of Environmental Medicine, New York University School of Medicine, New York, New York, United States of America; 2 Laboratory of Molecular Immunology, National Heart, Lung, and Blood Institute, National Institutes of Health, Bethesda, Maryland, United States of America; 3 Department of Biology, The Johns Hopkins University, Baltimore, Maryland, United States of America; 4 Department of Developmental Biology and Genetics, Stanford University School of Medicine, Stanford, California, United States of America; National Institutes of Health, United States of America

## Abstract

Polycomb group (PcG) proteins are key chromatin regulators implicated in multiple processes including embryonic development, tissue homeostasis, genomic imprinting, X-chromosome inactivation, and germ cell differentiation. The PcG proteins recognize target genomic loci through *cis* DNA sequences known as Polycomb Response Elements (PREs), which are well characterized in *Drosophila*. However, mammalian PREs have been elusive until two groups reported putative mammalian PREs recently. Consistent with the existence of mammalian PREs, here we report the identification and characterization of a potential PRE from human T cells. The putative human PRE has enriched binding of PcG proteins, and such binding is dependent on a key PcG component SUZ12. We demonstrate that the putative human PRE carries both genetic and molecular features of *Drosophila* PRE in transgenic flies, implying that not only the *trans* PcG proteins but also certain features of the *cis* PREs are conserved between mammals and *Drosophila*.

## Introduction

Polycomb group (PcG) proteins, together with the functionally antagonizing trithorax group proteins (TrxG), maintain a pre-determined state of transcription, which constitutes a cellular memory stable over many cell divisions [1], [2], [3], [4], [5], [6], [7], [8]. The PcG proteins act in at least two distinct but interacting protein complexes in mammals, Polycomb Repressive Complex 1 (PRC1, containing BMI1, RING1A, RING1B, CBX, and PHC) and PRC2 (EZH2, SUZ12, and EED) [9], [10]. The core complex of PRC1 in *Drosophila* consists of Polycomb (Pc), Polyhomeotic (Ph), Posterior sex combs (Psc), and *Drosophila* Ring (dRing) [11]; while PRC2 contains Enhancer of Zeste [E(z)], Extra sex combs, Suppressor of Zeste 12 [Su(z)12], Nurf55, and several other components [12]. In *Drosophila*, two additional PcG complexes were identified as Pho repressive complex (PhoRC, containing DNA binding proteins Pho/Phol and dSfmbt) and Polycomb repressive deubiquitinase (PR-DUB) [13], [14]. But the mammalian functional counterparts for PhoRC and PR-DUB remain unclear [15], [16]. The PcG complexes may use multiple mechanisms to silence transcription, for example, by making the chromatin more compact [17], or by interfering with transcription initiation [18], [19] and/or elongation [20]. It is generally agreed that PcG complexes employ epigenetic mechanisms that alter chromatin state to repress gene expression. A widely accepted model of PcG action is initiated by the PRC2 complex, which contains an enzymatic component EZH2 to trimethylate histone H3 at Lysine 27 (H3K27me3) [21], [22], [23], [24]. The methylated histone recruits PRC1, which binds to H3K27me3 through the chromodomain of the PC (Polycomb) protein [25], [26], leading to nucleation of the entire PcG complex. Although PRC2 and PRC1 should have overlapping binding sites according to this model, other studies revealed some exceptions, suggesting that PRC1 and PRC2 may have independent functions [27], [28]. In addition to the histone methyl-transferase activity of EZH2 in the PRC2 complex, the RING1B protein in the PRC1 complex acts as an E3 ubiquitin ligase, which ubiquitinates histone H2A at Lysine119 (H2AK119ub) [29]. The H2AK119ub may affect transcription by blocking efficient elongation [20]. In contrast to the transcriptional repressive activity of the PcG complex, the active H3K4me3 mark is generated by the TrxG complex [2], [30], [31] whose function opposes the PcG function.

Since PcG complex acts through regulation of chromatin structure, it is important to understand how they are recruited to chromatin, in order to characterize the molecular mechanism of PcG-mediated gene silencing. In *Drosophila*, the PcG proteins are recruited by sequence-specific DNA binding factors, such as PHO (homolog of YY1 [19]), GAF, PSQ, Zeste, and DSP1 [11], [32], [33], [34], [35], [36], to their target sites known as Polycomb Response Elements (PRE) to silence transcription of target genes [37], [38]. Although several target genes of the PcG proteins have been identified in mammals [39], [40], [41], mammalian PREs have remained elusive until recently [42], [43], partially due to the fact that recruiters such as GAF, PSQ, and Zeste are not conserved in vertebrates. Although two recent studies that identify mammalian PREs [42], [43] show the function of YY1 (homolog of Drosophila PHO) to be important, previous studies have not revealed many regions where YY1 and PcG proteins colocalize [44]. This suggests that the recruitment of mammalian PcG to their target sites may use mechanisms other than YY1. Therefore it is important to identify more functional mammalian PRE to study PcG recruitment *in vivo*. The lack of knowledge of the *cis* regulatory elements, the PREs, has hindered our understanding of the critical PcG regulation during mammalian development.

## Results

### PRC1 and PRC2 proteins have differential binding to the three DNA elements tested

**Figure 1:**
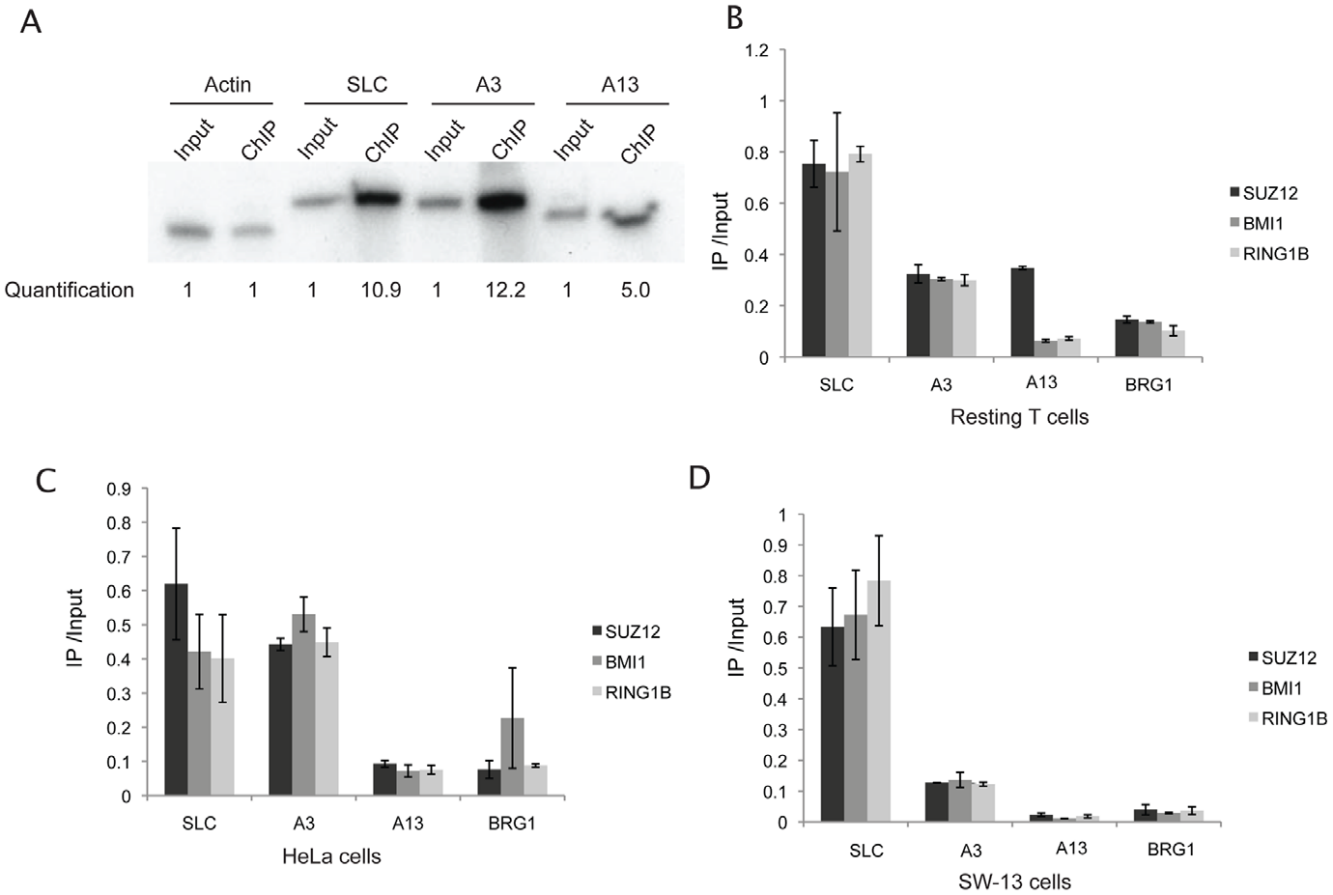
PcG proteins bind to the potential PREs in human cells. (**A**) Assessment of H3K27me3 levels at SLC, A3, and A13 regions using PCR. H3K27me3 ChIP DNA samples from resting T cells and their input controls were analyzed using ^32^P-labeled specific primers. Actin was used as control. Band intensities were quantified using Phospho Imager and indicated below the panel. (**B, C, D**) PcG proteins SUZ12, BMI1, and RING1B are enriched at the SLC and A3 regions compared to the A13 region in resting T cells (**B**), HeLa cells (**C**), and SW-13 cells (**D**), respectively. ChIP assays were performed using antibodies specific for SUZ12, BMI1, and RING1B with chromatin prepared from CD4^+^ T cells, HeLa cells and SW-13 cells. The ChIP DNA was analyzed by qPCR using primers specific for the SLC, A3 and A13 regions (primer sequences in Table 1).

In *Drosophila*, PcG proteins have enriched binding at PRE sites [15], [45], [46], [47]. Therefore, in this study we sought to identify potential PREs using a candidate approach based on the hypothesis that a functional PRE is associated with H3K27me3 and PcG proteins, and is localized near silenced genes. The first candidate we chose was an H3K27me3-enriched region (SLC) downstream of the *SLC17A7* gene [48], which encodes a sodium-dependent inorganic phosphate co-transporter [49] and is silent in T cells [48]. Since *Hox* genes are potentially regulated by PcG proteins, we also selected two regions (A3 and A13) from the *HoxA* gene locus (Table 1). Using ChIP-PCR (chromatin immunoprecipitation followed by PCR using isotope labeled primers) assays, we found that SLC, A3 and A13 displayed differential levels of H3K27me3 binding (Fig. 1A). To test for enrichment of PRC1 and PRC2 components at these loci, we used human resting CD4^+^ T cells to perform ChIP experiments using antibodies against PRC1 components BMI1 and RING1B, as well as PRC2 component SUZ12. Our data indicated that the three DNA elements SLC, A3 and A13 had differential binding of PcG proteins. The SLC element was highly enriched with all three PcG proteins: SUZ12, BMI1 and RING1B. The A3 element was associated with intermediate levels of all three PcG proteins, whereas relatively low PcG protein binding was detected at the A13 region (Fig. 1B). Similar results were obtained from HeLa cells (Fig. 1C) and SW-13 cells (Fig. 1D).

**Table 1:** Genomic coordinates of the 3 kb human putative PRE regions and sequences of specific primers used in ChIP experiments for each of these regions (sequence information is based on the UCSC hg18 assembly).

PRE Locus	Closest gene	Distance from TSS (Kb)	Chr:Genomic position
SLC	SLC17A7	12	19:54623621–54626731
NPR	NPR1	1	1:151916871v151919849
NeuD	NEUROD2	8	17:35009354–35011885
A3	HOXA3	8	7:27128850–27131636
XKR	XKR6	274	8:10821434–10824457
PITX	PITX3	14	10:103976617–103979186
BCAN	BCAN	12	1:154891106–154893906
UNC	UNC5A	−67	5:176103122–176106145
SND	SND1	513	7:127592544–127595536
BRG	BRG1	−12	19:10920061–10923101
A13	HOXA13	−1	7:27207068–27210086

### The PRE-mediated transcriptional repression is dependent on normal function of PcG proteins

**Figure 2:**
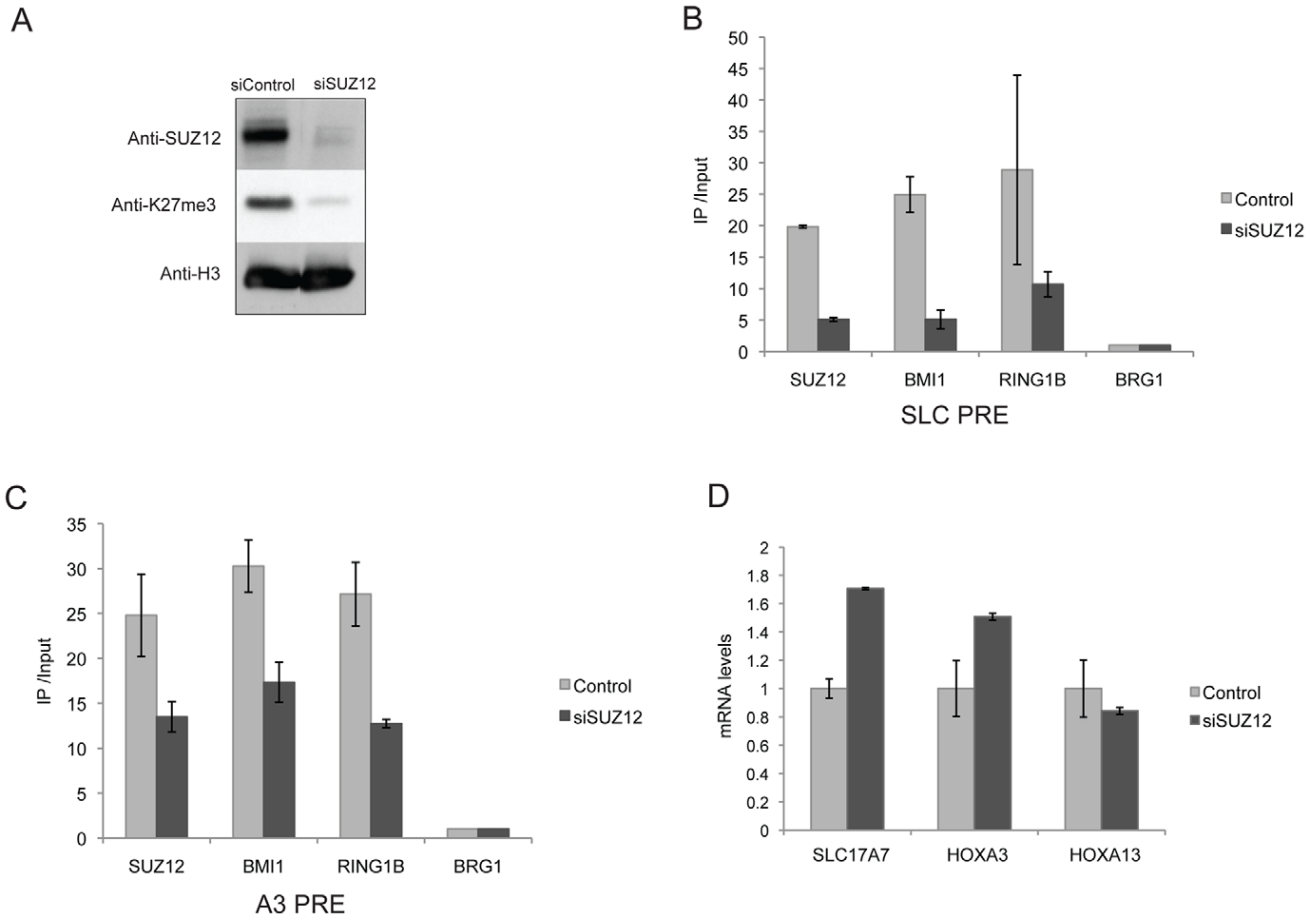
Normal PcG protein activities are required for the PRE-mediated transcriptional repression. Knocking down SUZ12 decreased the binding of PRC1 proteins at the endogenous SLC (**A**) and endogenous A3 (**B**) regions. ChIP assays were performed using the indicated antibodies, with chromatin from HeLa cells transfected with pREP4-Puro-siSUZ12 or the control vector. ChIP DNA was analyzed by qPCR using primers specific for the SLC-PRE and A3-PRE regions (Table 1). The specificity of ChIP experiment was confirmed by evaluating PcG binding at a region upstream of the *BRG1* gene locus, which showed very low level of PcG proteins in both the control and SUZ12 knockdown cells. (**C**) Knocking down SUZ12 in HeLa cells increased the expression of the endogenous *SLC17A7* (SLC locus) and *HoxA3* (A3 locus) genes but not the *HoxA13* (A13 locus) gene. Total RNAs were isolated from HeLa cells transfected with pREP4-Puro-siSUZ12 or a control vector and selected with puromycin. The expression level of the genes was determined by qRT-PCR analysis.

To examine whether the enrichment of H3K27me3 and PcG proteins at the putative PREs requires normal function of PcG proteins, we knocked down SUZ12, an essential component of the PRC2 complex [50], in cell culture system. As shown in Figure S1, the siRNA construct targeting SUZ12 sequences decreased the protein level by over 80%. Global H3K27me3 level was also significantly reduced, probably due to the important function of SUZ12 in PRC2 complex to generate the H3K27me3 modification [51]. We then analyzed the level of PcG proteins at the endogenous SLC and A3 regions using ChIP assays. Consistent with the reduction in its global expression level, SUZ12 binding at both SLC and A3 regions decreased significantly in the SUZ12 knockdown cells (Fig. 2A and 2B). The binding of PRC1 proteins BMI1 and RING1B were also significantly reduced, consistent with the idea that the H3K27me3 mark generated by the PRC2 complex is required for recruitment of the PRC1 complex to the potential PRE sites [19]. In summary, in SUZ12 knockdown cells, the chromatin state at both the SLC and A3 putative PREs changed from high PcG binding and activity to low PcG binding and activity.

To investigate whether these changes at the chromatin level affect expression of endogenous genes near the SLC and A3 loci, we examined the mRNA levels of the genes at the vicinity (Table 1). Interestingly, in HeLa cells, knockdown of SUZ12 resulted in an increased expression level of *SLC17A7* (near SLC element) and *HoxA*3 (near A3 element). In contrast, no obvious increase of *HoxA13* (near A13 element) expression was observed (Fig. 2). Together, these results suggested that normal function of PcG proteins is required for the repressive activities of the putative human PREs.

### The putative human PREs repress reporter gene expression in *Drosophila*

**Figure 3:**
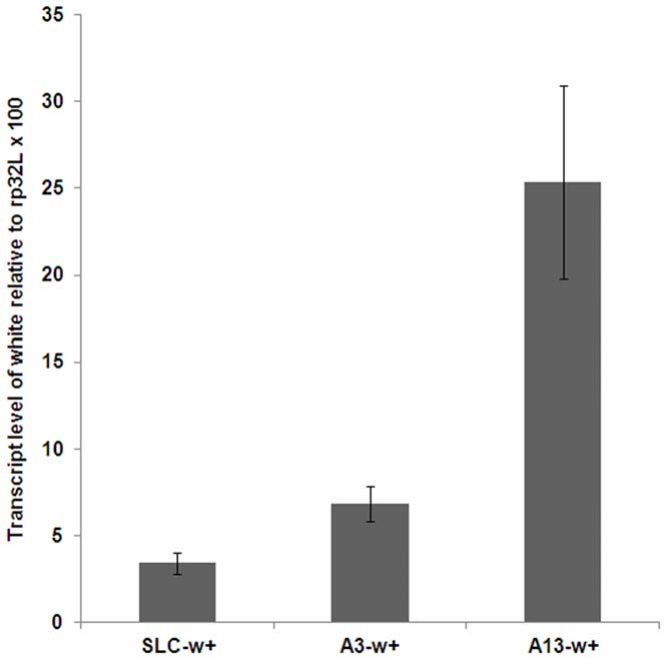
The putative human PREs repress reporter gene expression in *Drosophila*. Quantification of the *white* gene expression controlled by putative human PREs. mRNA of *white* was quantified by qRT-PCR and normalized to a constitutively expressed gene *rp32L* transcript level, followed by multiplying with a factor of 100. For each transgenic line, the qRT-PCR (*white/rp32L*) data is obtained from 2–3 qPCR reactions and averaged. And for each human DNA element, the data is the average of 5–6 independent lines and the error bars indicate standard error from all independent lines tested.

**Figure 4:**
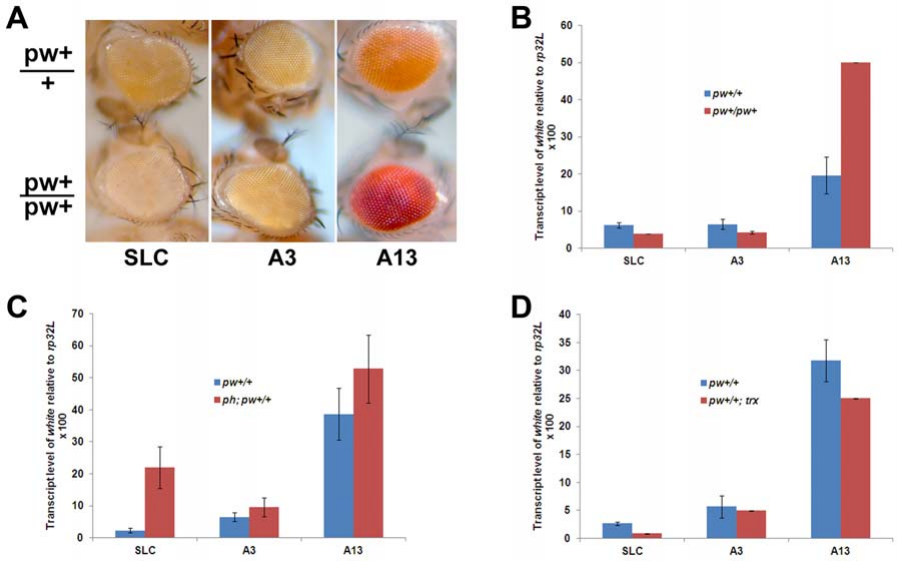
The putative human PREs have characteristics resembling *Drosophila* PREs in transgenic flies. (**A**) Pairing-sensitive silencing of human PREs: The same transgene in heterozygous (upper panels) or homozygous (lower panels) flies. (**B**) Quantification of results shown in (**A**) by qRT-PCR analyses. 2–3 PCR reactions were performed for each genotype. (**C**) Derepression of *miniwhite* transcription by a mutation in the *ph* gene. Quantification of *white* gene transcript from the same *miniwhite* transgene at either a *wild-type* background or the *ph (ph^401^)* mutant background by qRT-PCR analyses. 2–3 PCR reactions were performed for each genotype. (**D**) Repression of *miniwhite* transcription by a mutation in the *trx* gene. Quantification of *white* gene transcript from the same *miniwhite* transgene in a temperature-sensitive *trx (trx^1^)* background at either the permissive temperature or the restrictive temperature by qRT-PCR analyses. 2–3 PCR reactions were performed for each genotype.

To investigate the functional conservation of the putative human PREs, we tested their activities in *Drosophila* by assaying the PRE-mediated silencing effect on the *miniwhite* reporter gene expression in adult fly eyes. In this experiment, a 3-kb genomic DNA fragment surrounding the SLC, A3 or A13 region was placed next to the *miniwhite* gene (within 100-bp) individually in the pCasper3 expression vector (Table 1, Table 2, and Figure S2). Each of these reporter constructs was incorporated as a single transgene into the *w^67c23^* fly genome, which has a deletion of the promoter region of the endogenous *white* gene and is a transcript-null allele ([52], Flybase and data not shown). To better control the eye color difference in males *vs.* females (males usually have darker eye color than females even if the transgene is on autosomes), we used males for all experiments in Figures 3 and 4. The silencing effect was then evaluated by examining the eye color of male flies and by measuring the *white* mRNA levels with quantitative RT-PCR (qRT-PCR). The qRT-PCR is a more direct and quantitative method to monitor the transcriptional levels of the *white* gene. In this experiment, newly enclosed heterozygous males (0–1 day old in adulthood) were used for RNA extraction and quantification using an internal control *rp32L*, a constitutively expressed gene. To rule out the positional effect in gene expression, 5–6 independent transgenic lines were generated from each construct and the results obtained from all lines were analyzed and shown in Figure 3. The repressive effects of *miniwhite* reporter gene expression by the putative human PRE elements in *Drosophila* eyes correlated well with their binding affinity with PcG proteins and repressive activities in human cells (Fig. 1 and 2). The SLC element, which was a strong PRE candidate in human cells, had the strongest silencing effect in the *Drosophila* reporter assay (Fig. 3). The A3 element showed modest repressive activity in the *Drosophila* assay (Fig. 3), consistent with its moderate PcG protein binding in human cells (Fig. 1B and 1C). In contrast, the A13 region did not show any obvious repression activity in *Drosophila* (Fig. 3), consistent with its lack of PcG binding in human cells (Fig. 1B and 1C).

**Table 2:** Summary of the repression of *miniwhite* gene expression by human PREs in all the transgenic flies.

Potential PRE	*white* expression in independent lines				Pair-sensitive repression	Derepression by *PcG*
SLC	line1	2nd	−1	−		
	line2	X	−4	−−−−	yes	
	line3	3rd	−2	−−	no	
	line4	3rd	−1	−	no	
	line5	2nd	−4	−−−−	yes	derepressed by *Ph*
	average		−2.4			
NPR	line1	2nd	−3	−−−		
	line2	2nd	1	+	yes	derepressed by *Ph*
	line3	2nd	−2	−−	yes	
	line4	2nd	−3	−−−	no	
	line5	2nd	−4	−−−−	yes	
	average		−2.2			
NeuD	line1	2nd	1	+	yes	
	line2	2nd	−1	−	yes	derepressed by *Ph*
	line3	2nd	−3	−−−	no	
	line4	3rd	−2	−−	no	
	line5	2nd	−3	−−−	yes	
	line6	3rd	−4	−−−−	yes	derepressed by *Ph*
	average		−2			
A3	line1	X	1	+		
	line2	3rd	−3	−−−	yes	derepressed by *Ph*
	line3	2nd	−2	−−	yes	derepressed by *Ph*
	line4	X	−3	−−−	yes	
	line5	X	2	++	yes	
	average		−1			
XKR	line1	2nd	1	+	no	
	line2	2nd	1	+	yes	derepressed by *Ph*
	line3	2nd	−3	−−−	yes	
	line4	3rd	−1	−	no	
	line5	2nd	−2	−−	no	
	average		−0.8			
PITX	line1	2nd	−1	−	no	
	line2	2nd	1	+	yes	
	line3	2nd	1	+	no	
	line4	2nd	−4	−−−−	yes	derepressed by *Ph*
	line5	2nd	−1	−	no	
	average		−0.8			
BCAN	line1	3rd	2	++	no	
	line2	3rd	2	++	no	
	line3	2nd	−3	−−−	yes	derepressed by *Ph*
	line4	X	−2	−−		
	line5	2nd	1	+	yes	
	average		0			
UNC	line1	3rd	2	++	no	
	line2	3rd	1	+	no	
	line3	3rd	−2	−−	no	
	line4	3rd	3	+++	no	
	line5	3rd	−1	−	no	
	line6	3rd	1	+	no	
	average		0.67			
SND	line1	2nd	−3	−−−		
	line2	3rd	2	++	yes	
	line3	3rd	−1	−	yes	
	line4	2nd	−2	−−	no	
	line5	2nd	1	+	no	
	line6	2nd	−2	−−		
	average		0.83			
A13	line1	3rd	4	++++	no	
	line2	2nd	3	+++	no	
	line3	2nd	2	++	no	
	line4	3rd	2	++	no	
	line5	3rd	1	+	no	
	line6		3	+++		
	average		2.5			
BRG	line1		2	++		
	line2		2	++		
	line3		2	++		
	average		2			

Eleven potential human PRE elements were individually tested for their suppression of *white* gene expression, judged by fly eye color with the corresponding transgene; pair-sensitivity and de-repression by *ph^401^* mutation. The eye color was classified to eight levels from −4 to +4, where −4 was the palest and +4 the darkest. On average 5–6 independent lines were tested for each transgene and the results were summarized. The strongest group contains SLC, NPR, LGR and NeuD; the intermediate group is consisted of A3, XKR, PITX and BCAN; and the weakest group includes PDE, UNC and A13. BRG serves as a control.

As the White protein is required for eye pigmentation, the eye color of flies reflects the White protein level and activity. Consistent with the order of the *white* transcript levels in SLC, A3 and A13 driving transgenes (Fig. 3), the eye color of the corresponding transgenic heterozygous flies ranged from light color for the SLC and A3 transgenes to a much darker color for the A13 transgene (Fig. 4A, upper panels).

A characteristic phenomenon for PRE-mediated gene silencing in flies is the pairing-sensitive silencing (PSS), in which the PRE silencing effect is enhanced in the homozygotes for the transgene. The PSS effect has been suggested as a result of dimerization of PcG proteins at the paired PRE loci [53]. Because of the extremely light eye color of the SLC flies, we used 3-day old males for experiments in Figure 4A–B. As shown by eye color of transgenic flies with an autosomal transgene (Fig. 4A) and the quantification of the *white* gene mRNA levels (Fig. 4B), the SLC-driving transgene showed the strongest PSS, in which the homozygous eye is lighter than the heterozygous one (Fig. 4A) and by quantification, the *white* transcript reduced ∼40% in homozygotes compared to heterozygotes (Fig. 4B). The A3 human PRE elements showed weaker PSS effect, in which the homozygous eye color is about the same as the heterozygous one (Fig. 4A). In contrast, the A13 element exhibited an approximately 2-fold increase of *miniwhite* expression in homozygous flies, typical of non-repressive *cis*-acting sequences. In addition, eye color variegation, often associated with PRE-mediated repression, was also apparent for SLC (Fig. 4A).

To confirm that the silencing of the *miniwhite* reporter gene by the human PREs in *Drosophila* is mediated by PcG proteins, we crossed the same human PRE-conjugated *miniwhite* transgenes (on the second chromosome) to a *ph* (*polyhomeotic*) mutant background. Ph is one of the four core components of PRC1 complex in flies [7], [11]. We then analyzed the eye color and *white* transcript level using newly enclosed males (0–1 day old). Consistent with the involvement of PcG in silencing, the *ph^401^* mutation (on the X chromosome) relieved SLC-mediated repression 9.6-fold (Fig. 4C). A derepression of 1.5-fold was observed for the moderate repressor A3 (Fig. 4C). In contrast, there was no significant derepression for the non-repressor A13 (Fig. 4C).

Transcriptional repression by the PcG silencing machinery is counteracted by the action of the TrxG protein complex [7]. To test whether mutations in TrxG proteins have an opposite effect compared to the *ph* mutation, we crossed the same PRE-conjugated *miniwhite* transgene (on the second chromosome) to a *trx* temperature sensitive allele *trx^1^* (on the third chromosome) [54]. By shifting the larvae to restrictive temperature at 29°C, we then analyzed the eye color and *white* transcript level in newly enclosed males (0–1 day old). We observed a 3.1-fold repression of the *miniwhite* expression in the SLC line in the *trx^1^* background at restrictive temperature compared to the same transgene at permissive temperature at 25°C (Fig. 4D). However, such repression is not significant for either the moderate repressor A3 or the non-repressor A13 (Fig. 4D).

### *Drosophila* PcG proteins have selective binding affinity to the putative human PREs

**Figure 5:**
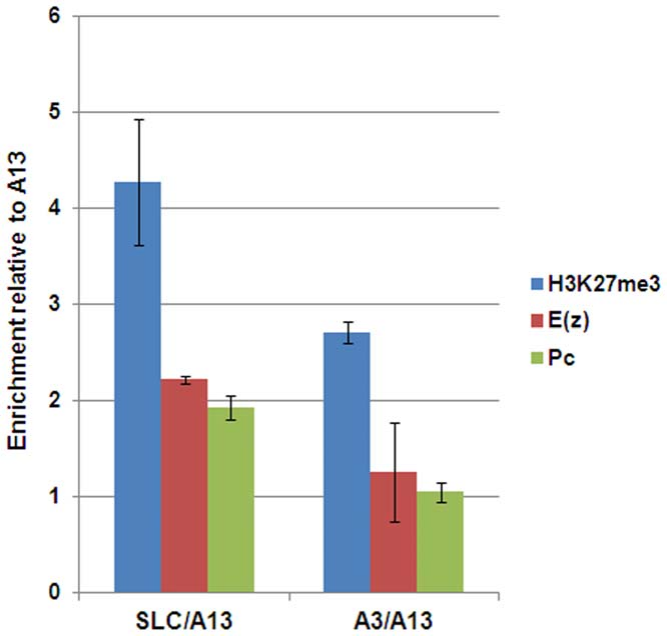
The putative human PRE SLC element has enriched *Drosophila* PcG protein binding in transgenic flies. The H3K27me3 modification and *Drosophila* PcG proteins are enriched at the SLC region compared to the A13 region. The H3K27me3 modification is also enriched at the A3 region compared to the A13 region, but no enrichment of *Drosophila* PcG proteins has been detected at the A3 region. ChIP assays were performed using antibodies specific for H3K27me3, E(z), and Pc with chromatin prepared from fly heads. The ChIPed DNA was analyzed by qPCR using primers specific for either the SLC or the A3 region and normalized to the A13 region in the same ChIP experiment. 2–3 independent ChIP experiments were performed for each antibody and three qPCR reactions were performed for each region in every ChIP experiment.

We next examined the relative enrichment of the H3K27me3 modification and PcG proteins at the putative human PREs in transgenic flies. To have a better internal control for ChIP experiments, we generated fly strains that either has P[*SLC-w^+^*]; P[*A13-w^+^*] or P[*A3-w^+^*]; P[*A13-w^+^*] dual transgenes. As shown in Figure 5, ChIP with the anti-H3K27me3 antibody using the P[*SLC-w^+^*]; P[*A13-w^+^*] strain enriched the SLC region 4.3-fold compared to the A13 region. Using the P[*A3-w^+^*]; P[*A13-w^+^*] strain to ChIP with anti-H3K27me3, the A3 region was also enriched 2.7-fold relative to the A13 region. Likewise, ChIP with antibodies against the PRC1 component Pc or the PRC2 component E(z) enriched SLC region sequence about 2-fold compared to the A13 region. However, this enrichment of Pc and E(z) was not significant at the A3 region, consistent with its less effective PRE activity. Therefore, we concluded that the stronger PRE activities of the SLC element were associated with higher levels of the H3K27me3 modification and the binding of both PRC1 and PRC2 proteins.

## Discussion

In this study, we demonstrated that the PcG-enriched DNA *cis*-elements in human primary CD4^+^ T cells have roles in repressing transcription of neighboring genes, and such repressive activities depend on normal function of the *trans*-acting PcG proteins. Despite high homology of PcG proteins among different organisms [55], it is unclear to which extent the *cis*-acting elements, namely the PREs, are also conserved during evolution. Interestingly, we showed that the PcG-enriched sequences not only repress transcription in human cells, but also carry this ability to repress reporter gene expression in *Drosophila*. Recent research has suggested other molecular mechanisms that target PcG proteins to specific genomic loci in mammalian cells, such as the non-coding RNAs [56] and the pRB family proteins [57]. Identification of more functional PREs in mammals will allow for sequence comparison and functional analysis to explore whether transcription factor-mediated recruitment ensures PcG-PRE interaction in mammals, which is still under a lot of debates [10].

Recently, two other groups also reported identification of human PREs in mouse [42] and human [43], which revolutionarily changed the view of the molecular mechanisms underlying PcG function in mammals. Interestingly, in one of these reports [42], it was also shown that the mouse PRE can repress reporter gene expression in *Drosophila*, and such repressive function can be further modified by mutations in *Drosophila* PcG genes, which is probably due to the fact that mouse PRE can recruit fly PcG proteins. These data are highly consistent with what we report here. PcG proteins have been found to play multiple roles in stem cell maintenance and tumorigenesis in mammals. Several key developmental regulators are associated with PRC complexes as well as H3K27me3 modification in human and mouse embryonic stem cells [40], [41], [58] and in human embryonic fibroblasts [59]. Failures in PcG function have profound effect on diseases, such as cancers and tissue dystrophy [60]. Therefore, understanding the mode of action of PcG proteins is essential for understanding mammalian development and PcG dys-regulations during pathological processes. Our successful identification of genomic regions that mediate PcG-dependent transcriptional repression demonstrates evidence for the existence of human counterparts of *Drosophila* PREs and provides an opportunity for further characterization of the PcG targeting mechanisms in mammalian cells. Studying potential defects by deleting these elements in mouse will be the next step to definitely establish their functional roles as mammalian PREs during development.

## Materials and Methods

### Human T cell isolation

Human resting T cells were purified from the whole blood using the lymphocyte separation medium (Mediatech) and Pan T-cell isolation kit II (Miltenyi Biotech) as described previously [61]. The T cells were from healthy donors through the blood bank of National Institutes of Health and do not require any IRB and consent.

### RNA isolation and quantitative PCR

Total RNAs from human cells were isolated as described previously [62]. Total RNAs from fly heads were extracted using TRIzol reagent (Cat#15596) according to manufacturer's suggestion (Life Technologies Inc.). The equivalent of 0.5-head was used per PCR reaction. cDNA was synthesized using SuperScript III RNase H reverse transcriptase (Invitrogen). To quantify gene expression levels, real-time PCR was carried out with primers and TaqMan probes from Applied Biosystems Inc. using the Universal RT-PCR Master Mix (Cat# 4309169, Applied Biosystems Inc.). Each PCR reaction was performed in duplicates or triplicates and the Ct numbers for each reaction were collected. Quantification was carried out by the absolute quantification method using standard curves.

### ChIP Assays

ChIP assays using human cells (SW-13 cells are obtained according to [63], Hela cells were obtained from ATCC, Inc.) were performed as described previously [62]. The antibodies used were histone H3K27me3 (Upstate, 07-449), SUZ12 (Abcam, ab12201), BMI1 (Upstate, 05-637), and RING1B (Abcam, ab3832). Quantification of the ChIP samples from human cells was carried out by the comparative Ct method [64]. Briefly, the target sequences in the ChIP and the input DNA samples were amplified with primers specific for the potential PRE regions or the control regions and the fold difference between the ChIP and the input DNA were calculated. As a control for the ChIP experiment a locus upstream of the human *BRG1* gene which showed very little enrichment of H3K27me3 was used.

ChIP assays using the transgenic flies were performed as described previously [54] except the following changes. About 20 fly heads were isolated from a strain with double transgenes P[*SLC- w^+^*];P[*A13- w^+^*] or P[*A3-w^+^*]; P[*A13-w^+^*]. The equivalent of 1.5-head was used per PCR reaction per antibody. The following amounts of antibodies were used: 5 µl anti-H3K27me3 (Upstate, 07-449), 2.5 µl anti-E(z), and 2.5 µl anti-Pc (from R. Jones and R. Kingston, respectively). For quantification of ChIP DNA samples, input DNA, mock precipitated DNA (no antibody) and ChIP DNA with specific antibodies were all analyzed by real-time PCR using the primers obtained from Integrated DNA Technologies. The ChIP and mock DNA were normalized with the input DNA amount. The values from ChIP DNA were further corrected by subtraction of the non-specific signal derived from the mock precipitate (ChIP DNA- mock DNA)/Input, and compared with each other.

### RNA interference

For silencing human *SUZ12*, the target sequence from the *SUZ12* cDNA (GGACCTACGTTGCAGTTCACT; position 1053–1073) was inserted into pBS-U6 vector. An unrelated sequence was used as control. The cloned *SUZ12* sequence and the control sequence along with the U6 promoter were then subcloned into pREP4-puro as described previously [65]. For RNA interference analysis, HeLa cells (ATCC, Inc.) were transfected with the siRNAs or control and selected with 2 µg of puromycin/ml for 72 hours.

### Fly strains and husbandry

Flies were raised on standard cornmeal molasses agar medium at 25°C unless stated otherwise. The *w, ph^401^* and *trx^1^* strains were obtained from the Bloomington Stock Center (stock numbers are BL-5392 and BL-2114, respectively).

### Generation and analyses of transgenic flies with different human PRE elements

Potential human PREs elements were subcloned into the multi-cloning sites within 100-bp from the *white* gene in a pCasper3 expression vector. Each plasmid bearing either a potential human PRE or a control element was introduced into the *w^67c23^* fly genome *via* standard P-element-mediated transformation. The *w^67c23^* (*w*) mutation deletes the promoter and the first exon region including the start codon of *white* gene, therefore represents a null allele of *white* ([52] and Flybase). On average, 5–6 independent transgenic lines were generated and analyzed for each construct. To test for the pairing-sensitive silencing effect, a double-balanced stock was generated for each transgenic strain with a 2^nd^ or 3^rd^- chromosomal insertion (Cyo used as the 2^nd^ chromosomal balancer and TM6B used as the 3^rd^ chromosomal balancer). The stock was self-crossed and crossed with the parental *w* strain simultaneously. The resulting homozygotes and heterozygotes (with no balancer) were compared with each other. To test the derepression of *miniwhite* by *ph^401^*, male flies with the autosomal transgenes were crossed to virgin females either from a *w*, *ph^401^* strain, or a *w* strain. Newly enclosed (0–1 day old) male progenies from each cross were compared with each other. To test the repression of *miniwhite* by the temperature-sensitive *trx^1^* allele, double-balanced males with the second-chromosomal transgenes were crossed to virgin females from a *w*, *trx^1^* strain. The resulting *w*; *P[w^+^]/+*; *trx^1^/TM6B* males were backcrossed to the *w*, *trx^1^* strain to obtain *w*; *P[w^+^]/+*; *trx^1^* males. The larvae were shifted to restrictive temperature at 29°C and newly enclosed (0–1 day old) males were obtained and compared with newly enclosed males at permissive temperature at 25°C.

### ChIP-qPCR primers

SLC-Forward AACCCTGCACTGGGAAAAAA

SLC-Reverse AAGTCACAGAATCCCATGAAAGG

SLC-TaqMan Probe ACCCCTGGCTCCTGCCCCATT

A3-Forward CATAGCGGATCTTTCTGGAATGA

A3-Reverse CCATGAGCAAGGTGGACTCA

A3-TaqMan Probe ATTGAGAGGCAAAGTGCAGGATGG

A13-Forward CCTGCAGGATCCAGACCAA

A13-Reverse GGTCAGGACAAATCCAGGATCA

A13- TaqMan Probe CTGGGCTTGGGCTTTTATCTG

BRG1-Forward GCAGGAGAATCGCTTGAACCT

BRG1-Reverse CTTGTTTTTTGAGACAGAGTCTCACTCT

BRG1-TaqMan Probe TGCAGTGAGCCAAGATCTCGACA

## Supporting Information

Figure S1 **SUZ12 small interference RNA inhibits SUZ12 expression and the H3K27me3 signals in HeLa cells.** HeLa cells were transfected with pREP4-Puro-siSUZ12 or a control vector with an unrelated sequence and analyzed using Western blotting with antibodies against SUZ12, H3K27me3. Histone H3 was used as loading control.
(PDF)

Figure S2 **Index of eye color averaged from 3–6 independent transgenic lines for each of the 12 human PRE tested for repressing** ***white*** **gene expression in** ***Drosophila*** **.** −4 is for the lightest eye color and +4 is for the darkest eye color, all data are from Table 2.
(PDF)
