# Supplementary figures and images for: A Novel Human Polycomb Binding Site Acts As a Functional Polycomb Response Element in Drosophila
Source: PLoS One. 2012 May 3;7(5):e36365. doi: 10.1371/journal.pone.0036365 (PMC3343078; doi:10.1371/journal.pone.0036365)

**Figure S1**

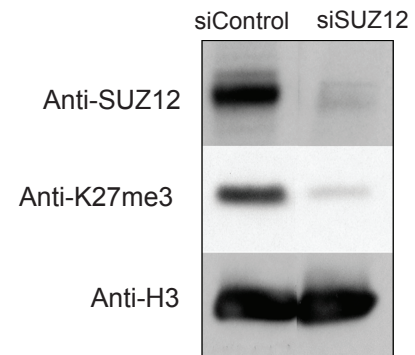

Supplement: Figure S1 — SUZ12 small interference RNA inhibits SUZ12 expression and the H3K27me3 signals in HeLa cells. HeLa cells were transfected with pREP4-Puro-siSUZ12 or a control vector with an unrelated sequence and analyzed using Western blotting with antibodies against SUZ12, H3K27me3. Histone H3 was used as loading control. (PDF) [file pone.0036365.s001.pdf]

Figure S2

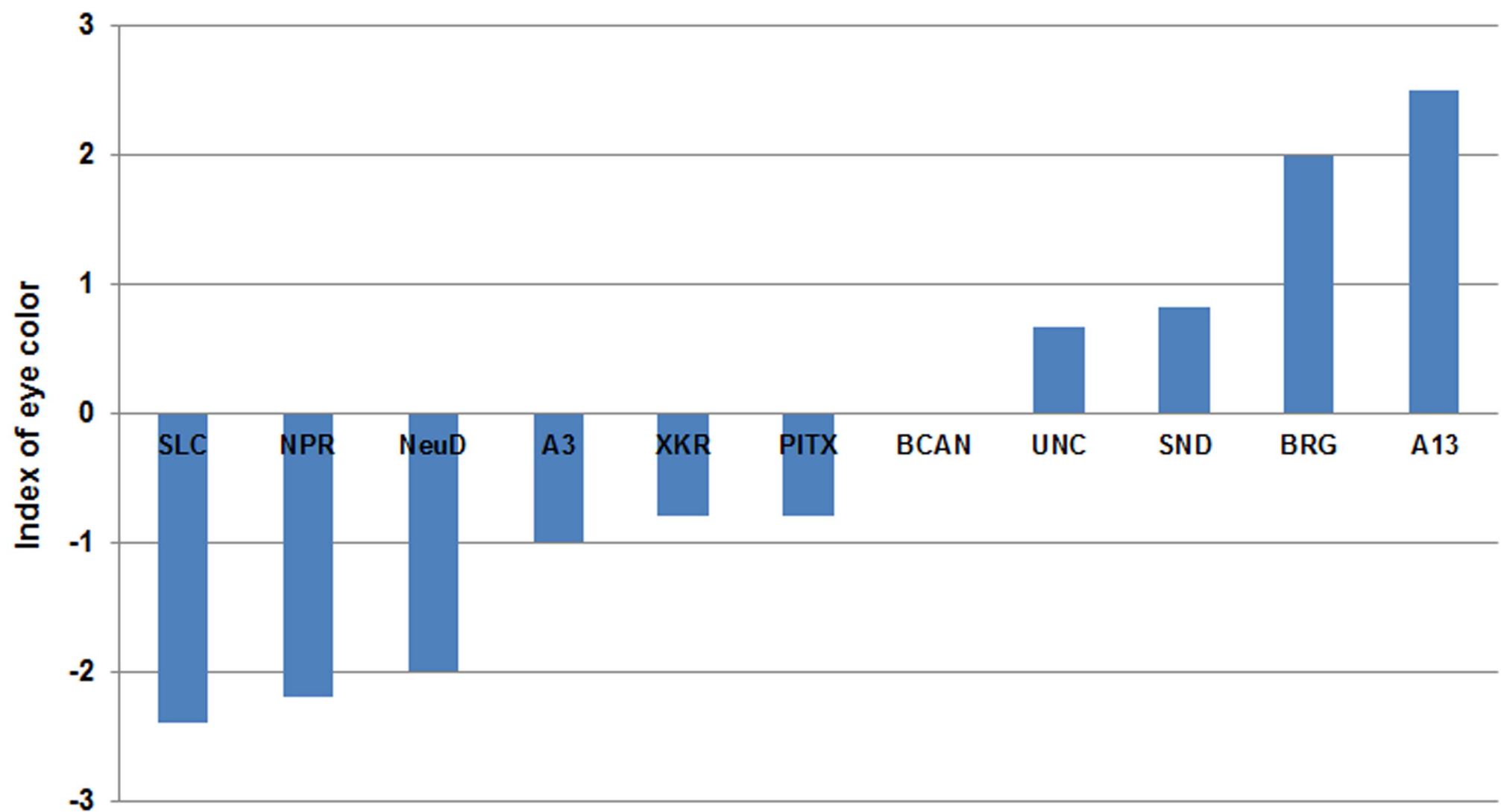

Supplement: Figure S2 — Index of eye color averaged from 3–6 independent transgenic lines for each of the 12 human PRE tested for repressing white gene expression in Drosophila . −4 is for the lightest eye color and +4 is for the darkest eye color, all data are from Table 2. (PDF) [file pone.0036365.s002.pdf]
